# Carotenoids and lipid production from *Rhodosporidium toruloides* cultured in tea waste hydrolysate

**DOI:** 10.1186/s13068-020-01712-0

**Published:** 2020-04-16

**Authors:** Feng Qi, Peijie Shen, Rongfei Hu, Ting Xue, Xianzhang Jiang, Lina Qin, Youqiang Chen, Jianzhong Huang

**Affiliations:** 1 Engineering Research Center of Industrial Microbiology of Ministry of Education, College of Life Sciences, Fujian Normal University, Fuzhou, 350117 Fujian China; 2 Medicine and Products of the State Oceanic Administration, Fujian Key Laboratory of Special Marine Bioresource Sustainable Utilization, College of Life Sciences, Fujian Normal University, Fuzhou, China

**Keywords:** *R. toruloides*, Tea waste hydrolysate, Torularhodin, Torulene, Lipid

## Abstract

**Background:**

In this study, renewable tea waste hydrolysate was used as a sole carbon source for carotenoids and lipid production. A novel *Rhodosporidium toruloides* mutant strain, RM18, was isolated through atmospheric and room-temperature plasma mutagenesis and continuous domestication in tea waste hydrolysate from *R. toruloides* ACCC20341.

**Results:**

RM18 produced a larger biomass and more carotenoids and α-linolenic acid compared with the control strain cultured in tea waste hydrolysate. The highest yields of torularhodin (481.92 μg/g DCW) and torulene (501 μg/g DCW) from RM18 cultured in tea waste hydrolysate were 12.86- and 1.5-fold higher, respectively, than that of the control strain. In addition, α-linolenic acid production from RM18 in TWH accounted for 5.5% of total lipids, which was 1.58 times more than that of the control strain. Transcriptomic profiling indicated that enhanced central metabolism and terpene biosynthesis led to improved carotenoids production, whereas aromatic amino acid synthesis and DNA damage checkpoint and sensing were probably relevant to tea waste hydrolysate tolerance.

**Conclusion:**

Tea waste is suitable for the hydrolysis of microbial cell culture mediums. The *R. toruloides* mutant RM18 showed considerable carotenoids and lipid production cultured in tea waste hydrolysate, which makes it viable for industrial applications.

## Background

Tea is one of the oldest and most widely consumed aromatic beverages and has immense economic and medicinal value globally [1]. Since the early twentieth century, the annual global consumption of tea has reached millions of tons. According to statistics from the FAO, China and India are the largest tea producers and consumers, with the total tea production in China reaching 1.75 million tons in 2012 with continuous increasing growth, accounting for 38% of global total tea production. In addition to consuming tea as a beverage, a large amount of tea leaves are processed into instant tea powder and tea polyphenols using a method aimed at extracting water-soluble substances [2]. Thus, various renewable tea waste products are produced as a result of tea leaf extraction processes.

The cell wall of tea leaves comprises cellulose, hemicellulose, lignin, tannins, and structural proteins. The conventional treatment approach for utilizing tea waste was to convert it into biofuels, feedstock for domestic animals or for use as crop fertilizer, both of which have been implemented for many years [2, 3]. However, the traditional conversion of tea waste into feedstock and fertilizer cannot meet the needs of the rapidly accumulating tea waste, which has gradually become a threat to the environment and a burden to tea processing industries. The environmental and cost-effective utilization of tea waste has therefore gained significant interest and additional attention in related studies.

Dilute acid hydrolysis has been an efficient, economical, and the most commonly used method for the pretreatment of lignocellulose because acids penetrate lignin without any preliminary biomass pretreatment and then break down cross-linked lignin, cellulose, and hemicellulose biopolymers in order to yield various sugar molecules [4, 5]. Glucose, xylose, arabinose, and acetate are released from lignocellulosic hydrolysate, which can be used as a sole carbon source for microbial cultivation to produce target compounds [6–8]. Compared with lignocellulosic biomass, tea leaves can be hydrolyzed easily for microbial cell culture mediums because of the significantly lower levels of lignin present in tea leaves [9]. This results in a small amount of byproduct, polyaromatic compounds, being generated from the acid hydrolysis of lignin in the hydrolysate. However, few studies to date have addressed the utilization of tea waste hydrolysate for microbial cultivation [10].

**Fig. 1:**
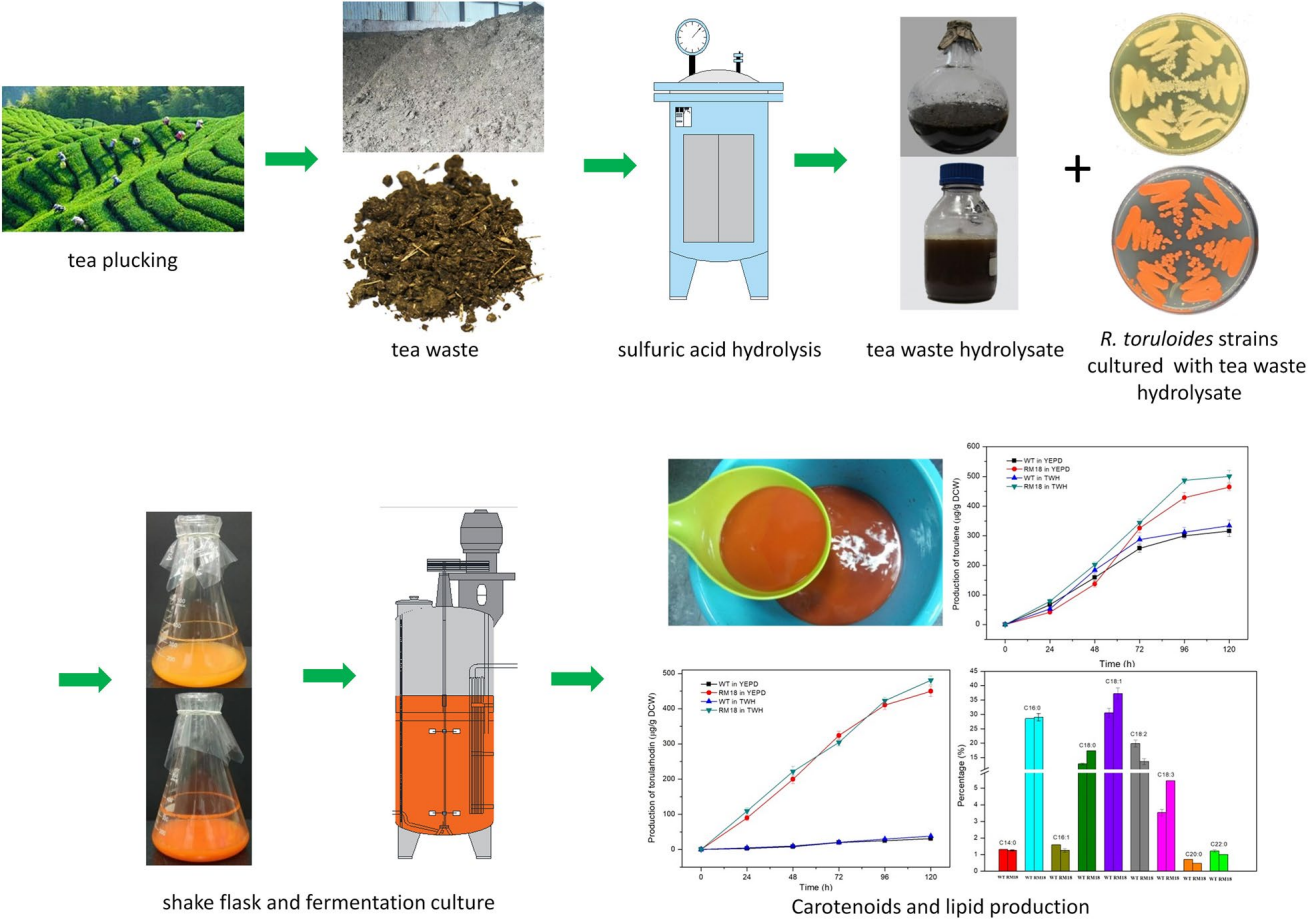
The process of *R. toruloides* cultivation using tea waste hydrolysate for carotenoids and lipid production in this study

Currently, increased attention is being paid to microbial lipids produced from oleaginous bacteria, yeasts, fungi, and microalgae because of their multiple advantages and analogous properties compared with those derived from typical animal grease and plant oils [11, 12]. *Rhodosporidium toruloides* is a promising oleaginous yeast strain that has been considered for potential application in microbial lipid production [13, 14]. The red, nonpathogenic *R. toruloides* strain can accumulate lipids to above 50% of dry cell weight (DCW) using a wide variety of carbon sources, and its lipid production has been studied in batch and fed-batch culture [5, 15, 16]. Additionally, *R. toruloides* is a natural producer of carotenoids, including β-carotene, torulene, and torularhodin, which are valuable molecules in manufacturing processes, e.g., in chemical, pharmaceutical, feed, and cosmetics industries [17]. β-carotene is a precursor of vitamin A and has antioxidant properties, making it an extremely important industrial compound [18]. Torulene and torularhodin have strong anti-oxidative properties owing to their 13 double bonds [19]. In addition to their antioxidant functions, torulene and torularhodin have also been shown to have an effective and significant inhibiting impact on the growth of prostate cancer in mice, indicating that these two carotenoids are likely associated with tumor apoptosis [20]. In our previous work, *R. toruloides* mutant strains with a strong tolerance for the inhibitory lignocellulosic hydrolysate were obtained using atmospheric room temperature plasma (ARTP) mutagenesis [5, 21]. In this study, the *R. toruloides* mutant strains RM11, RM14, and RM18 were obtained through continuous domestication processes in tea waste hydrolysate (TWH) from their corresponding strains, M11, M14, and M18, respectively. The mutants RM11, RM14, and RM18 were able to grow and accumulate β-carotene, torulene, torularhodin, and lipids in TWH when TWH was used as the sole carbon source without prior detoxification (Fig. 1). Furthermore, high-throughput RNA sequencing (RNA-seq) was employed to analyze the different global transcriptome profiles between the mutant strains and wild-type *R. toruloides* ACCC 20341, thereby illustrating the possible genes and pathways that are involved in TWH tolerance and carotenoids and lipid biosynthesis.

## Results and discussion

### Tea waste and TWH

**Fig. 2:**
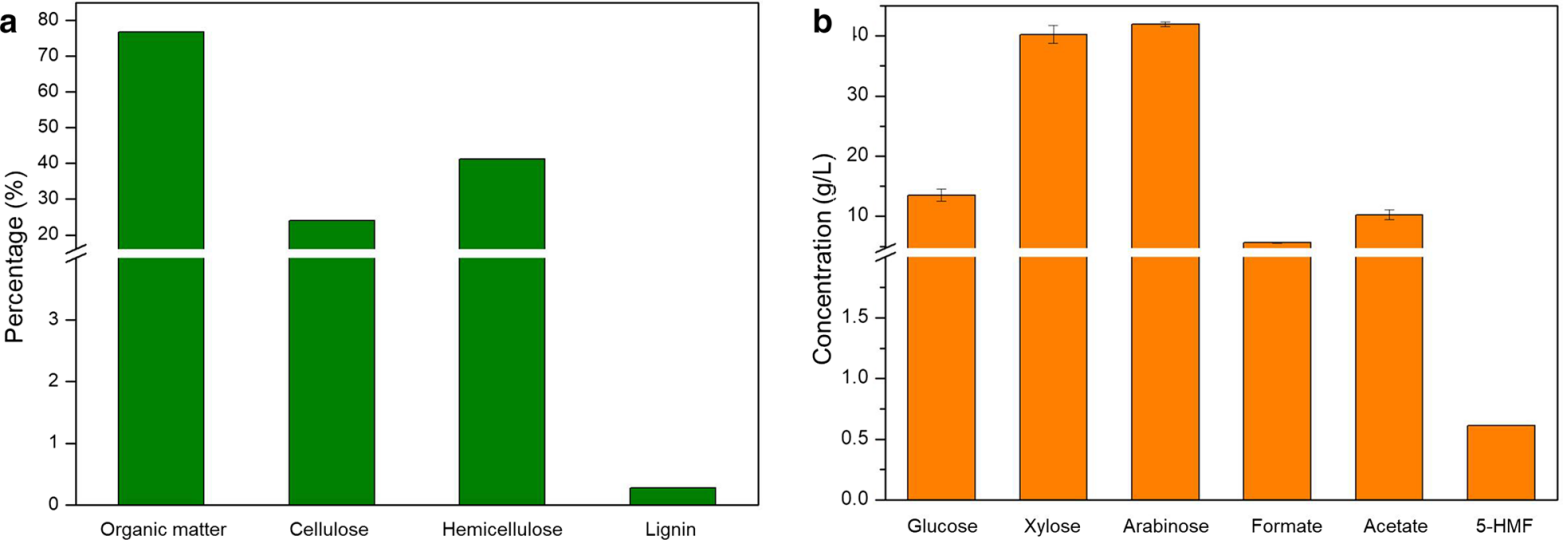
The contents of the tea waste used in this study (**a**), and the concentration of released compounds from tea waste hydrolysate (**b**)

The sample of tea waste treated after aqueous phase extraction was examined for quantification of organic content matter, cellulose, hemicellulose, and lignin by the Fujian Academy of Agricultural Sciences, China. Organic matter content is generally measured as organic C and/or total N content. The organic matter in tea waste was found to be roughly 76.81% (dry weight basis), among which 24.06% was cellulose and 40.2% was hemicellulose (Fig. 2a). The hemicellulose content in tea waste was greater than the cellulose content and was much higher than that in many other leaves and stalks [22]. As expected, the lignin content in tea waste was found to be very low, roughly 0.28% (dry weight basis) (Fig. 2a). Theoretically, there will be a relatively large amount of xylose and arabinose generated in TWH, but a very small amount of byproduct polyaromatic compounds due to the content of hemicellulose and lignin in tea waste, respectively. Generally, more than 200 tons of tea waste was generated each day after aqueous phase extraction of tea product by Fujian Xian Yang-yang Biotechnology Co., Ltd., which supplied us with the tea waste. These large amounts of tea waste can be a great sustainable resource for microbial cultivation. Figure 2b shows the released compounds from TWH pretreated by dilute sulfuric acid hydrolysis after concentrating TWH to ten times its original solution. The monosaccharides in the concentrated TWH were glucose, xylose, and arabinose, with concentrations of approximately 13.53, 40.21, and 41.89 g/L, respectively. Noticeably, the concentration of xylose and arabinose was much higher than that of glucose released from the TWH due to the presence of abundant hemicellulose in tea waste. Furthermore, the inhibitory acid-soluble lignin (polyaromatic) compound was always generated in lignocellulosic hydrolysate [4, 5]; however, almost no polyaromatic compounds were detected in TWH. Formate and acetate represented the primary inhibitory compounds, in addition to a small amount of 5-HMF (Fig. 2b). In fact, the released acetate could be utilized by *R. toruloides* strains, while all monosaccharides had been completely consumed. Therefore, TWH is more suitable as a carbon source than lignocellulosic hydrolysate.

### Isolation of *R. toruloides* RM11, RM14, and RM18

**Fig. 3:**
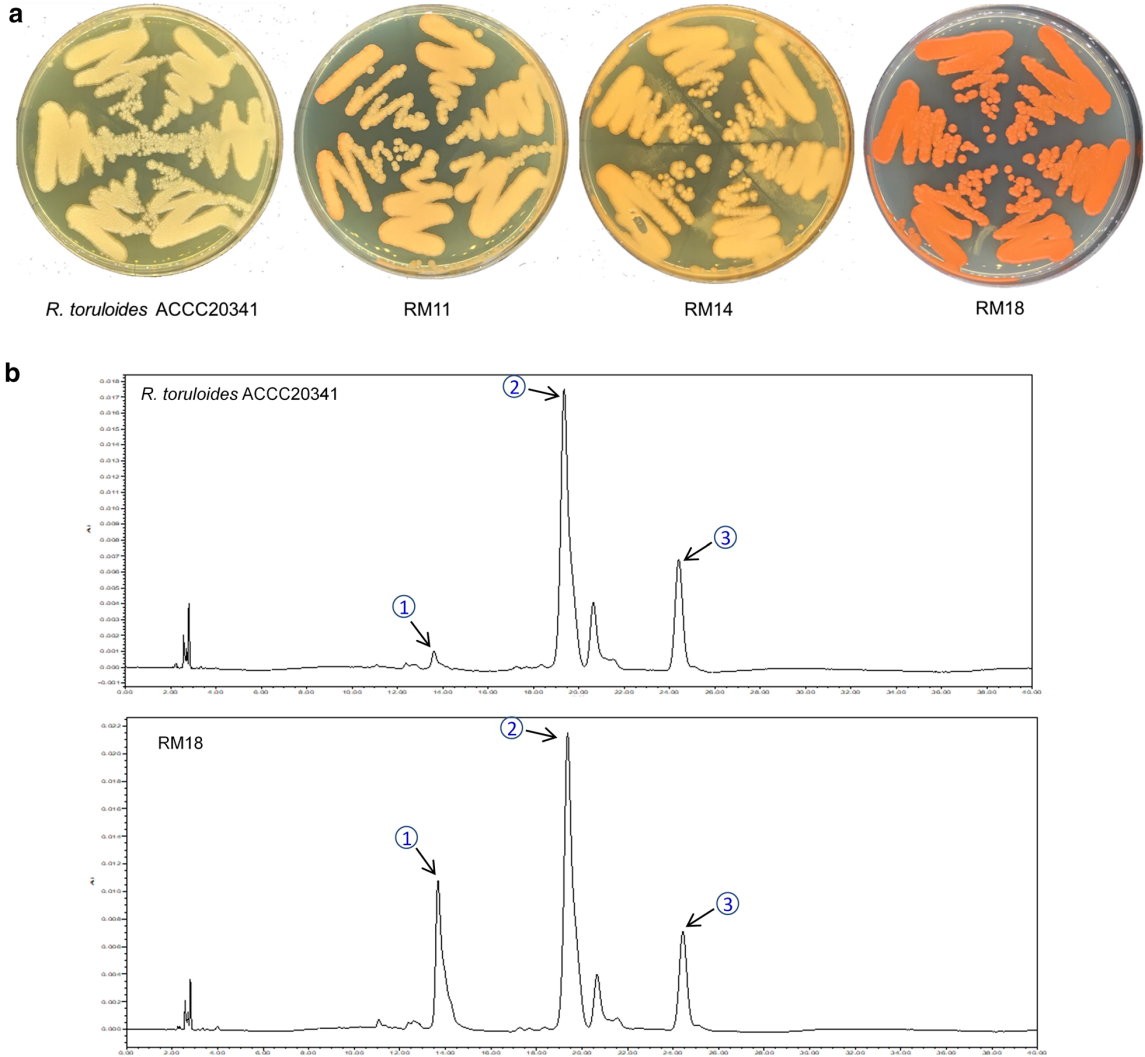
The isolated *R. toruloides* mutant strains RM11, RM14, and RM18 (**a**), and the identified carotenoids obtained from the three strains (**b**). The carotenoids peaks: 1, torularhodin; 2, torulene; 3. β-carotene

In this study, the lignocellulosic hydrolysate-tolerant *R. toruloides* strains M11, M14, and M18 obtained from our previous studies [5, 21] were selected following multiple rounds of adaptation and screening in TWH. Among all the tolerant mutants, the novel mutant strains with the highest biomass accumulation were isolated and named RM11, RM14, and RM18 through continuous domestication processes in TWH from their corresponding strains M11, M14, and M18, respectively (Fig. 3a). It can be inferred from Fig. 3a that RM18 accumulated more pigments than the other two strains. The extracted pigments were then analyzed using HPLC, and the results showed that torularhodin, torulene, and β-carotene accounted for the bulk of the pigment components (Fig. 3b). The presence of torularhodin and torulene has been described in many yeast and fungi, such as *Rhodotorula* sp., *Neurospora* sp., and *Sporobolomyces* sp. [23, 24]. In recent years, there have been increasing studies related to torularhodin and torulene biosynthesis and applications [25].

### Carotenoids production from *R. toruloides* strains in TWH

**Fig. 4:**
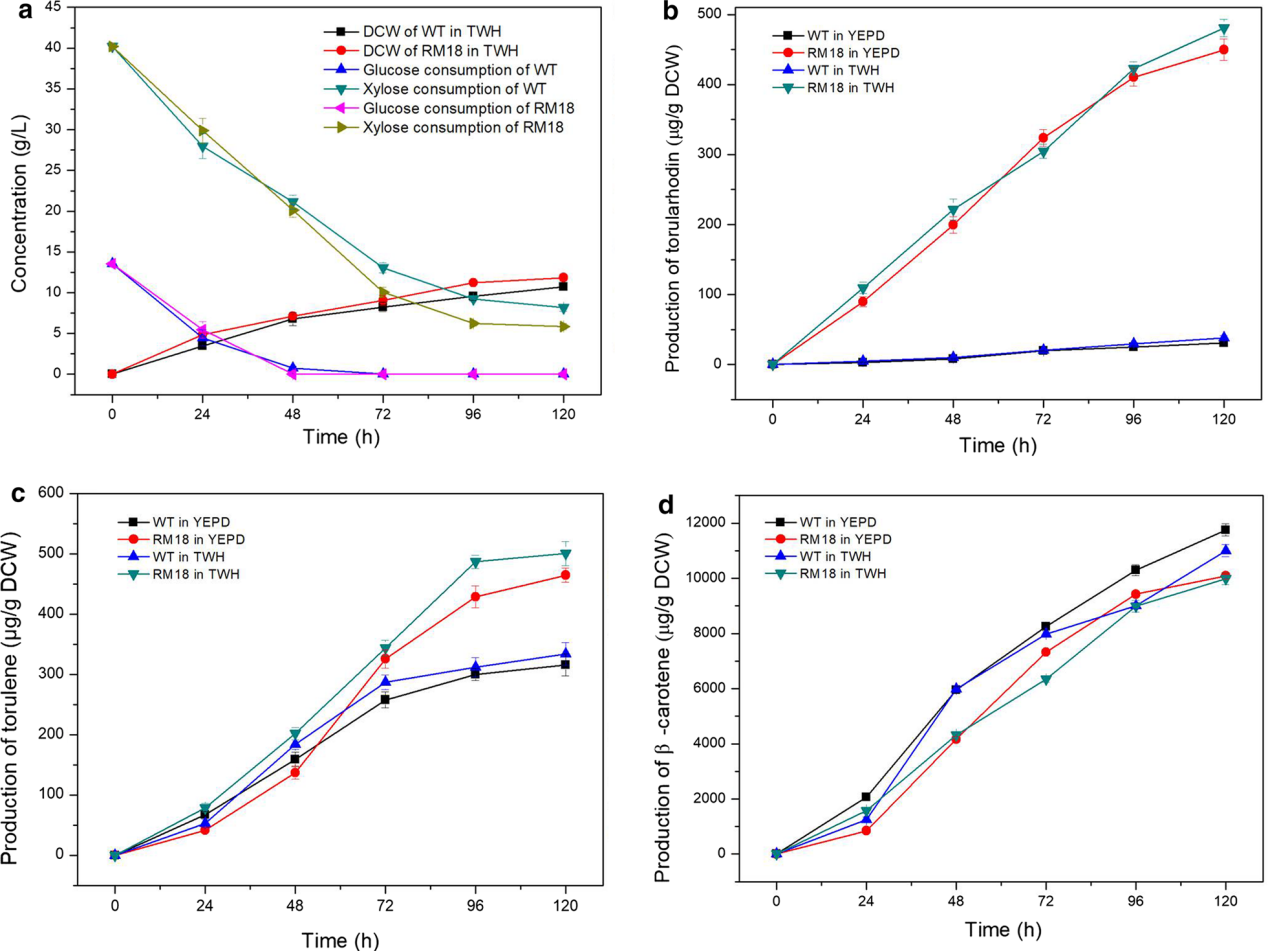
The cell growth, consumption of carbon sources, and carotenoids production by RM18 and the wild-type strain *R. toruloides* ACCC20341 used as the control cultured in TWH and YEPD. **a** Accumulation of biomass and consumption of xylose and glucose by RM18 and the control strain. **b** Production of torularhodin (μg/g DCW) by RM18 and the control strain. **c** Production of torulene (μg/g DCW) by RM18 and the control strain. **d** Production of β-carotene (μg/g DCW) by RM18 and the control strain. Data are represented as mean ± standard deviation (SD)

Among the three domesticated *R. toruloides* strains, RM18 has the highest yields of carotenoids and lipids according to the analysis (Table 1). Therefore, RM18 was selected as the candidate mutant strain in the subsequent study, and *R. toruloides* ACCC20341 was used as a control. Figure 4a shows cell growth and consumption of xylose and glucose in TWH by RM18 and the control. Glucose was quickly consumed within the first 48 h by each strain. Xylose was also quickly consumed from inoculation, and more than 80% xylose could be utilized at the end of cultivation, whereas arabinose could not be utilized at all. RM18 had a higher xylose utilization rate than the control, which likely led to a higher final biomass accumulation (11.85 g/L). As expected, RM18 accumulated significantly more carotenoids than the control. Interestingly, the highest yield of torularhodin from RM18 cultured in TWH was 481.92 μg/g DCW, which was 12.86-fold higher than that of the control strain (Fig. 4b). The torularhodin yields of RM18 and the control strain were slightly lower in the yeast extract–peptone–dextrose growth (YEPD) medium compared with both cultured in TWH. Similar results were observed for torulene yields (Fig. 4c). After 120 h of cultivation in TWH, the highest yield of torulene reached 501 μg/g DCW by RM18, which was 1.5-fold higher than that obtained by the control strain. Furthermore, both RM18 and the control strain could accumulate at least 5% more torularhodin and torulene in TWH compared with that accumulated when cultured in YEPD. Conversely, the β-carotene yield of RM18 (10.08 mg/g DCW) was significantly lower than that of the control strain (11.96 mg/g DCW) when TWH was used as the sole carbon source (Fig. 4d). Unexpectedly, no significantly enhanced β-carotene yield was found from either strain when cultured in TWH compared with that when cultured in YEPD.

**Table 1:** Carotenoids and lipid production by *R. toruloides* ACCC20341 and the three mutant strains cultured in TWH

Strains	Biomass (g/L)	Total pigment (mg/g)	Lipid titer (g/L)	Lipid content (%)
*R. toruloides* ACCC20341	10.75 ± 0.65	16.83 ± 0.08	4.78 ± 0.08	44.61 ± 2.73
RM11	10.16 ± 0.23	13.15 ± 0.11	4.52 ± 0.03	41.80 ± 1.95
RM14	8.66 ± 0.15	12.48 ± 0.05	3.78 ± 0.04	40.65 ± 3.21
RM18	11.85 ± 0.49	20.01 ± 0.09	5.01 ± 0.07	42.23 ± 2.96

It has been reported that torularhodin and torulene are mainly accumulated in yeasts of *Rhodotorula* and *Sporobolomyces* genera [23, 25]. Very few reports are available on torularhodin and torulene production from *Rhodosporidium* genera. In fact, the wild-type *Rhodosporidium toruloides* ACCC20341 can only produce 30.98 μg/g DCW of torularhodin; however, the highest torularhodin yield (450.06 μg/g DCW) can be achieved from the mutant RM18. When TWH was used as the sole carbon source, the torularhodin production of RM18 could be further increased to 481.92 μg/g DCW, the highest titer found in most yeast, with the exception of *Sporobolomyces* genera [23]. In addition, torulene yield of RM18 was also significantly increased compared to the control strain. In microorganisms, the primary and essential role of these carotenoids is protection against the negative influence of reactive forms of oxygen, radiation, and unfavorable environmental stress [24, 26]. Thus, carotenoids will be rapidly accumulated and in large amounts in RM18 due to its mutagenesis in the inhibitory and toxic lignocellulosic hydrolysate in first round screening. Furthermore, the titers of both torularhodin and torulene were significantly improved in both of the two strains cultured in TWH. Another explanation is that the culture condition of TWH, e.g., C/N ratio, was more favorable for torularhodin and torulene production. It has been reported that the maximum carotenoid pigments of torularhodin and β-carotene production (12.9 mg/L or 2.3 mg/g) were obtained at the C/N ratio of 20:1 from *Rhodotorula glutinis* [27]. In addition, it is noted that β-carotene biosynthesis was prominent among all the carotenoid pigments, but its yield was not enhanced in TWH. Although the biosynthesis of torularhodin, torulene, and β-carotene shared a common precursor, γ-carotene, the synthetic pathway of torularhodin and torulene was different with β-carotene. In other words, γ-carotene served as the metabolic branching point between torulene/torularhodin and β-carotene biosynthetic pathways. Thus, improved torularhodin and torulene production was obtained in TWH, leading to the direction of decreased carbon flux toward β-carotene biosynthesis.

### Lipid production from *R. toruloides* strains in TWH

**Fig. 5:**
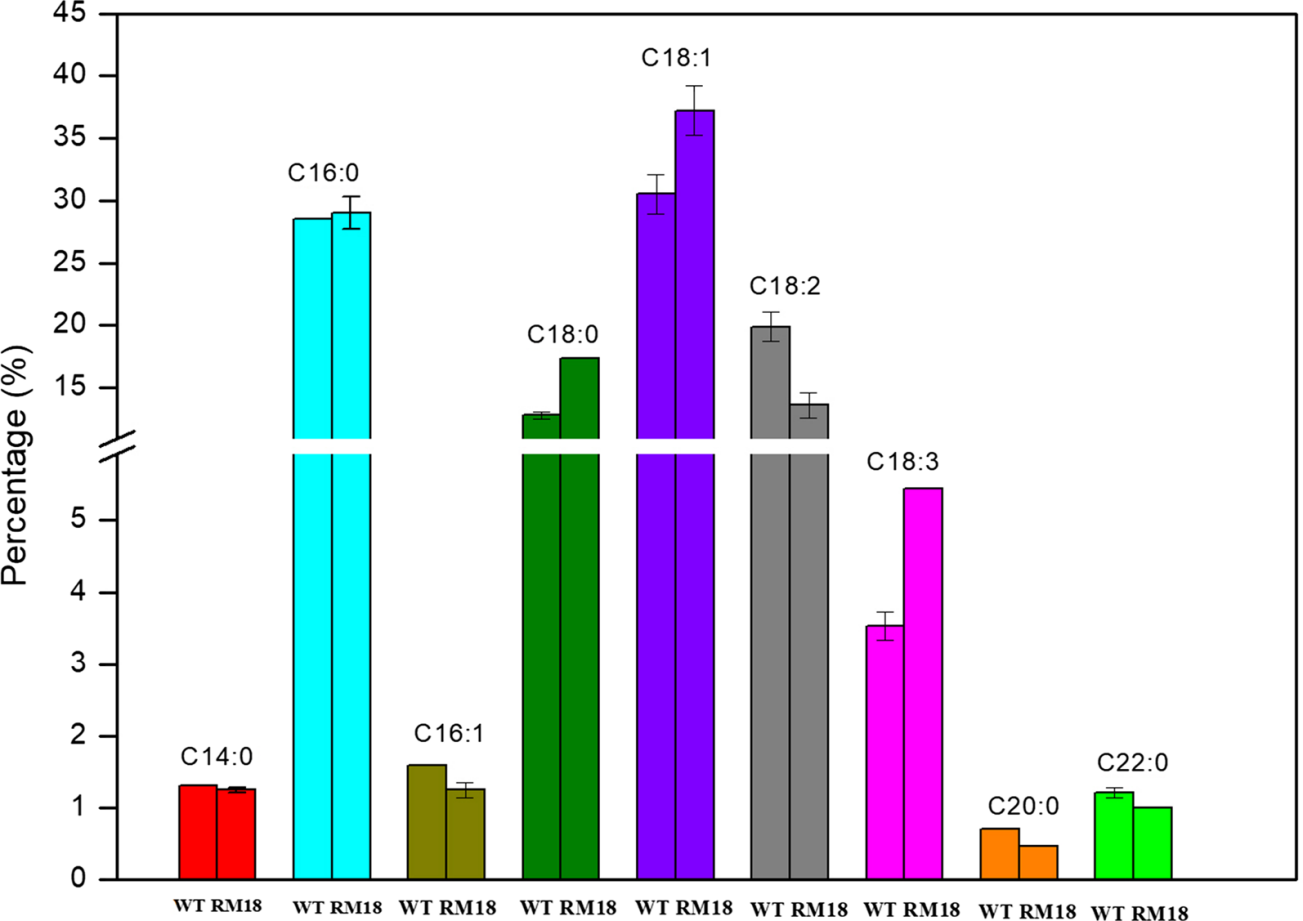
Fatty acid composition of the lipids produced by RM18 and the control strain cultured in TWH and YEPD. Data are represented as mean ± standard deviation (SD)

Lipid production from RM18 and the control strain using TWH as the sole carbon source were studied in this paper. RM18 and the control strain were able to accumulate lipids to 42.23% (5.01 g/L) and 44.61% (4.78 g/L) of DCW in TWH, respectively. The major fatty acid compositions of lipids produced by RM18 and the control strain cultured in TWH were identified as tetradecanoic acid (C14:0), palmitic acid (C16:0), palmitoleic acid (C16:1), stearic acid (C18:0), oleic acid (C18:1), linoleic acid (C18:2), α-linolenic acid (C18:3), eicosanoic acid (C20:0), and docosanoic acid (C20:0) (Fig. 5). The results showed that, for both strains, palmitic acid, stearic acid, oleic acid, and linoleic acid were the main components, accounting for more than 90% of total lipids, whereas the content of other fatty acids made up less than 5%. The production of stearic acid and oleic acid from RM18 was improved by 35.45% and 21.89% in TWH, compared to the control, while the production of linoleic acid in RM18 was lower than in the control strain. Among all the components of lipids derived from both *R. toruloides* strains, α-linolenic acid was identified in previous work [28]. α-linolenic acid is a highly valuable essential ω-3 (cisΔ9, 12, 15) polyunsaturated fatty acid, and is required for normal human growth and development [29]. It is noted that α-linolenic acid production from RM18 in TWH accounted for 5.5% of total lipids, which was 1.58 times more than that of the control strain. Thus, the additional enhanced production of α-linolenic acid from RM18 can be obtained in TWH. Here, we applied high-throughput RNA-seq to analyze the global metabolic responses to the stress in TWH, and to elucidate the differences of carotenoids and lipid biosynthesis between the RM18 and the control strain.

### Comparative analyses of transcriptomic profiling

**Fig. 6:**
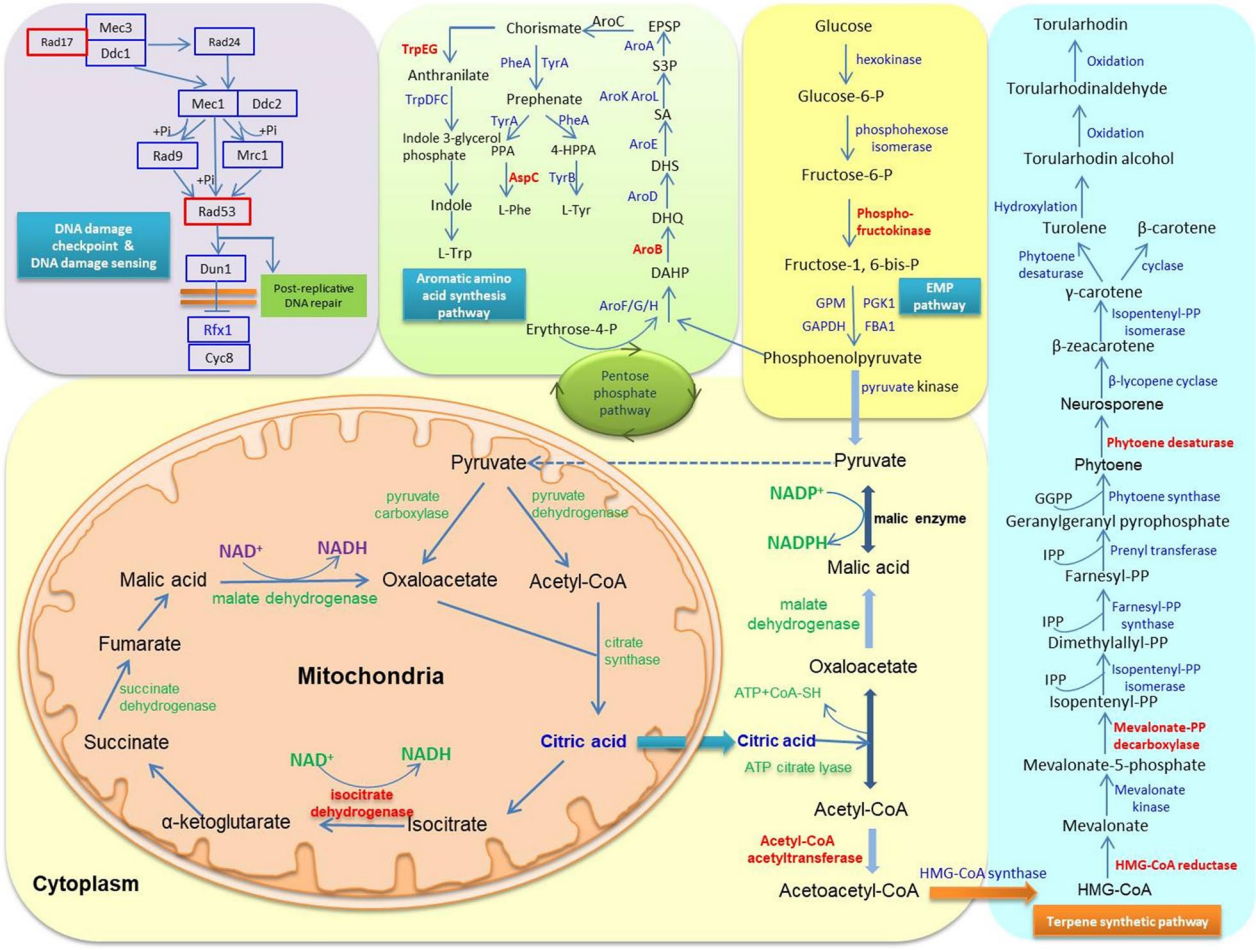
Reconstruction and illustration of the key genes with highly up-regulated transcriptional levels (≥ fivefold change) and the relevant cellular processes that the key genes are involved in. The highly up-regulated key genes involved in the pathway or cellular processes are highlighted

The transcriptome profiling of RM18 and wild-type *R. toruloides* at 12 h, 48 h, and 96 h was studied using RNA-seq via the Illumina platform. A total of 6595, 6530, and 6526 differentially expressed genes between RM18 and the wild type at lag phase, logarithmic phase, and stationary phase, respectively, were identified and annotated using the JGI and COG databases. With each sequence annotation and transcriptional profiling analysis, all the samples have the mapped ratio larger than 50% (Additional file 1: Table S1). Here, false discovery rates (FDR) < 0.01 and the value of fold change ≥ 2 were used to determine the statistical significance of gene transcription. Using a threshold of twofold change and gene ontology categories, we determined that 105 and 36 genes of the RM18 with the same transcriptional profiling in the three cell cycles were significantly up- and down-regulated, compared to the control strain, respectively. To validate the high-throughput RNA sequencing results, 20 genes were selected at random for quantitative PCR analysis (Additional file 1: Fig. S1). qPCR analysis was performed for the genes between the mutant RM18 and wild-type strain in all three phases. Good concordance was found between qPCR and RNA-Seq transcriptomics data (with correlation coefficient of 0.81). All genes with significantly changed transcriptional levels were divided into seven categories and nine cellular processes, according to the KEGG database. Among all the cellular processes, we found that several of these, such as the EMP pathway, tricarboxylic acid cycle, terpene synthesis pathway, shikimate pathway, and DNA damage sensing, were likely closely related to the mechanism of different carotenoids’ accumulation and tolerance for TWH between RM18 and the control strain. The reason for this was that these key genes involved in the five cellular processes shared a similar transcriptional pattern at lag, logarithmic, and stationary phases, with significantly high up-regulated values (≥ fivefold change) among the 105 genes (Fig. 6). The transcriptional levels of three genes coding for phosphofructokinase (PFK), acetyl-CoA acetyltransferase (ACAT2), and isocitrate dehydrogenase (IDH)—which are related to glycolysis and citric acid cycles—were found to be up-regulated at least five times in RM18, compared to the control strain (Fig. 6). The high upregulation of PFK in RM18 was considered to improve the EMP pathway. IDH catalyzed the dehydrogenation reaction of isocitrate through the reduction of NAD^+^ to NADH, while NADPH was regenerated from dehydrogenation of pyruvate by malic enzyme (ME1), the transcriptional level of which was also up-regulated 2.3 times in RM18, compared to the control strain. It was found that the increased intracellular NADH/NADPH availability improved biomass production [30], and is thought to be related to resistance against the inhibitory lignocellulosic hydrolysate [31]. Thus, upregulation of PFK, IDH, and ME1 in RM18 enhanced central metabolism functioning and NADH/NADPH availability, resulting in higher biomass accumulation, improved consumption of carbon sources in TWH, and higher tolerance for TWH, as confirmed in this study.

ACAT2 and HMG-CoA reductase (tHMGR) catalyzed the reactions of acetyl-CoA to acetoacetyl-CoA, and HMG-CoA to mevalonate, respectively, which represented the top portion of the mevalonate pathway. Increased carbon flux was suggested as being redirected toward the mevalonate pathway and terpenes biosynthesis pathway, due to the highly up-regulated ACAT2 and tHMGR in RM18, and mevalonate-PP decarboxylase (Fig. 6). The transcriptional level of phytoene desaturase (PDS) was up-regulated 8.03-fold in RM18, compared to the control strain, which will give rise to an additional improvement in flux toward γ-carotene biosynthesis. Theoretically, β-carotene and torularhodin/torulene yield from RM18 should be higher than that of the control strain, because γ-carotene is employed as a common precursor of β-carotene and torulene/torularhodin. Furthermore, there are no genes which are directly associated with torularhodin/torulene production found to be highly regulated in transcriptional level. The results showed that torularhodin and torulene yield from RM18 was more than 12 and 1.5 times higher than in the control strain, respectively. However, β-carotene yield of RM18 was significantly lower than in the control strain. It appears that most of the enhanced carbon flux and the portion originally used for β-carotene production had likely been contributed to torularhodin and torulene synthesis. Despite the considerable differences in torularhodin/torulene and β-carotene production between RM18 and the control strain, there were no significant transcriptional differences between the torularhodin/torulene and β-carotene synthesis pathway in either strain. We speculate that there is a likelihood that mutations occurred in the key genes involved in metabolic pathway reconstruction from γ-carotene to torularhodin biosynthesis, resulting in redirection of metabolic flux toward torularhodin/torulene biosynthesis.

Transcriptional levels of the genes coding AroB, AspC, and TrpEG in the aromatic amino acid synthesis pathway were found greatly up-regulated in RM18 compared to the control (Fig. 6). The shikimate pathway, tryptophan, and phenylalanine synthesis pathway were enhanced due to the three up-regulated genes. It has been reported that amino acid biosynthesis is related to the ethanol tolerance of microbial cells; for example, Zhao and Bai found that amino acid biosynthesis played an important role in ethanol tolerance and cell viability in yeast cells [32]. In addition, the up-regulated genes involved in aspartate, valine, isoleucine, and glutamine biosynthesis of *Aurantiochytrium* sp. contributed to inhibitory hydrolysate tolerance [7]. In the present study, the enhanced aromatic amino acid synthesis pathway likely led to the improved tolerance of RM18 against the stress of inhibitory hydrolysate and TWH, which is consistent with a previous report [33]. The mechanism for the correlation of aromatic amino acid synthesis and stress tolerance of microbial cells should be further studied. DNA damage sensing, and repairing and response mechanisms were thought to primarily suppress genomic instability in response to genotoxic stress [34].

The highly regulated genes coding Rad17 (6.13-fold) and Rad53 (5.49-fold) are involved in the cellular process of DNA damage checkpoint and sensing. The DNA damage checkpoint in the cell cycle is responsible for delaying DNA replication in response to genotoxic stress. Rad9, Rad17, and Rad24—responsible for DNA damage sensing and response—were found to be required for suppression of mutagenic post-replicative DNA repair during chronic DNA damage in *S. cerevisiae* [35]. Furthermore, DNA damage sensing and response can be initiated through the action of certain sensors, transducers, and effectors, which orchestrates the appropriate repair of DNA damage and resolution of DNA replication problems [34]. Thus, the enhanced cellular processes of DNA damage checkpoint and sensing in RM18 are activated and utilized for cell renovation from DNA damage, following ARTP mutagenesis and the continuous domestication processes in TWH.

## Conclusions

A novel *R. toruloides* mutant strain, RM18, was isolated through ARTP mutagenesis and domestication in TWH from its parental strain *R. toruloides* ACCC 20341. TWH was used as a sole carbon source. RM18 produced more carotenoids compared with the control cultured in TWH. Enhanced central metabolism and terpene biosynthesis led to improved carotenoids production, whereas aromatic amino acid synthesis and DNA damage checkpoint and sensing were shown to be relevant to TWH tolerance. RM18 exhibited more tolerance to TWH and indicated considerable carotenoids productivity, which is conducive for industrial use.

## Materials and methods

### Chemicals and reagents

All the chemicals and reagents used in this study for cell culture are analytically pure, while the reagents for HPLC analysis are chromatographic pure. Acetic acid, methanol and ethanol were supplied by Beijing Chemical Works (Beijing, China). Glucose, levulinic acid, arabinose, and 5-hydroxymethylfurfural (5-HMF) were purchased from Sigma-Aldrich (St. Louis, US). Xylose and vanillin were purchased from Amresco (Solon, US).

### Dilute acid hydrolysis of tea waste

The optimized procedure of acid hydrolysis of tea waste was modified and performed as described in a previous study [5]. The tea waste was supplied by Fujian Xian Yang-yang Biotechnology Co., Ltd. after aqueous phase extraction. More than 200 tons of tea waste per day has been generated from this company. Firstly, the tea waste was pretreated with 0.5% sulfuric acid (w/w) at the ratio of 10:1 (liquid-to-solid) at 121 °C for 40 min in a stainless autoclave with slightly mechanical stirring. Then the TWH was adjusted to pH 6.2–6.4 with calcium hydroxide, filtered, concentrated to 5–10 times the original, and composition analyzed by HPLC (Shimadzu LC-2030C, Japan). 10 g/L peptone used for nitrogen source was added in the prepared TWH.

### Strain and culture conditions

*Rhodosporidium toruloides* ACCC 20341 preserved in our lab was purchased from Agricultural Culture Collection of China (ACCC) and used as the wild-type strain in this study. *R. toruloides* M11, M14, M18 isolated from our previous works have tolerance for the inhibitory lignocellulosic hydrolysates [5]. In this work, *R. toruloides* RM11, RM14, and RM18 are obtained through continuous domestication using TWH from strains M11, M14, and M18, respectively. All the strains were grown in yeast extract peptone dextrose medium (YEPD, glucose 20/L, yeast extract 10/L, and peptone 20 g/L). Inoculum was grown in YEPD at pH 6.0, 30 °C, and 200 rpm in air-bath shaker until the cell density was near OD_600_ = 10. Then the culture was centrifuged at 20,000×*g* for 10 min, and the pellets were collected and suspended in TWH. After 96 h of incubation, the cell pellets were collected for carotenoids extraction and analysis. The nitrogen-limited TWH medium that was used for lipid production contained yeast extract 0.75 g/L (NH_4_)_2_SO_4_ 0.1 g/L, KH_2_PO_4_ 0.4 g/L in TWH.

### RNA isolation, high-throughput RNA-seq (HTR) library preparation

RNA isolation and RNA-seq library preparation were described in detail in the previous work [21]. Total RNA was isolated and purified using RNA purification kit (QIAGEN, Germany) according to the instructions. Agilent 2100 bioanalyser was utilized for examination of the quality of total RNA (rRNA 28 s/18 s). In order to avoid the transcriptional changes caused by the response to growth phases, 10 µg total RNA from lag phase (12 h), logarithmic phase (48 h), and stationary phase (96 h) from RM18 and the control strain were used for comparative analysis, respectively. All the qualified RNA samples were performed for RNA-seq library preparation and direct-sequencing on the Illumina platform. RNA libraries of single and paired-end were constructed. The whole procedure followed Illumina’s standard protocols and recommendations. Then annotation and data analysis were performed. Several alignment programs and database specifically for annotation of the *R. toruloides* transcriptomic data were employed, including KEGG and COG databases. The essential gene ontology terms were especially assigned by Blast2GO via a search of the NR database. The gene transcriptional patterns and the abundance of a particular transcript relative to controls are the desired information in this work. Data were determined through triplicate independent experiments.

### Quantitative real-time RT-PCR analysis

The RNA samples for quantitative PCR analysis were collected from cells grown under the same growth conditions as described in RNA-seq transcriptomic analysis. This analysis has been performed for verification of the high-throughput RNA-seq results. The method has been described in detail in the previous work [7]. The gene of GAPDH of *R. toruloides* was selected as control for normalizing expression of the samples. All the quantitative PCR reactions were carried out using the Mx3000P (Agilent Technologies, US) with SYBR fluorescence signal detection kit (Takara, Japan). The results obtained from the assay were converted into fold-changes using the formula:

$$2^{{ - \Delta \Delta C_{\text{t}} }} \left( { - \Delta \Delta C_{\text{t}} = \left[ {C_{{{\text{t}}({\text{target}})}} - C_{{{\text{t}}({\text{ref}})}} } \right]_{{{\text{M}}18}} - \left[ {C_{{{\text{t}}({\text{target}})}} - C_{{{\text{t}}({\text{ref}})}} } \right]_{\text{WT}} } \right),$$

where *C*_t_ represents the threshold cycle. Data were determined through triplicate independent experiments and statistical significance was considered at *P *< 0.05.

### Pigment and lipid extraction

Lipid extraction was performed when inoculum of each *R. toruloides* strain was grown in TWH at 30 °C, 220 rpm for 5 days. The cell samples were prepared and suspended in 5 mL 6 mol/L HCl at 80 °C for 1 h, and treated using 1 mL *n*-hexane combined with 0.75 mL absolute ethanol. The mixture was shaken vigorously, centrifuged at 12,000 rpm for 2 min, and collected. The extraction process was repeated 3 times. Solvents were evaporated under oxygen-free nitrogen on a heating block maintained at 50 °C. As for pigment extraction, the cell samples were first treated by yeast cell grinder (Sigma-Aldrich, China). Then the pigment was extracted using acetone with the solid–liquid ratio of 1:3. The mixture was also shaken vigorously and centrifuged at 13,000 rpm for 5 min. This extraction process was repeated 3 times, filtered, and prepared for HPLC analysis.

### Analysis methods

Cell density was measured using a UV–Vis spectrophotometer (Shimadzu, Japan). Quantitative analysis for compounds in TWH was performed using a Shimadzu 10AVP HPLC system (Shimadzu, Japan) equipped with a RID-20A refractive index detector, Aminex HPX-87H 300 × 7.8 mm column (Bio-Rad Laboratories, US) at 40 °C with 5 mM H_2_SO_4_ as the eluent with a flow rate of 0.5 mL/min. As for examination of carotenoids extracted from the *R. toruloides* strains, a UV spectrophotometry method was employed. Lipid analysis was carried out using an Agilent 7890A GC (Agilent Technologies, US) equipped with a CP-FFAP CB capillary column (25 m × 0.32 m × 0.30 μm). The column temperature was kept at 180 °C for 0.5 min and heated to 260 °C at 10 °C/min, then kept for 8 min. The temperatures of the injector and detector were set at 245 and 260 °C, respectively. The heptadecanoic acid methyl ester was used as the internal standard.

## Supplementary information

**Additional file 1: Table S1.** Sequencing quality evaluation of the cDNA samples of *R. toruloides* cultured in TWH at different phases. **Fig. S1.** Examination and verification of the transcriptional levels of the clustered genes with quantitative RT-PCR method. Data were determined through triplicate independent experiments and the error bars represent standard deviation.

## Data Availability

The datasets used and/or analyzed during the current study are included in this article and available from the corresponding author on reasonable request.

## Abbreviations

ARTP: Atmospheric room temperature plasma
TWH: Tea waste hydrolysate
5-HMF: 5-Hydroxymethylfurfural
COG: Clusters of orthologous groups of proteins
FDR: False discovery rates
aroB: 3-Dehydroquinate synthase
aroD: 3-Dehydroquinate dehydratase
aroK: Shikimate kinase 1
aroL: Shikimate kinase 2
tyrA: Prephenate dehydrogenase
pheA: Prephenate dehydratase
Aspc: Aspartate aminotransferase
TrpEG: Anthranilate synthase
Ddc1, 2: DNA damage checkpoint protein
Rad9, 17, 53, 24: DNA damage checkpoint proteins
Mec1: Serine/threonine-protein kinase
Mrc1: Mediator of replication checkpoint protein
Dun1: DNA damage response protein kinase
RFX1: Transcriptional regulator
Cyc8: General transcriptional corepressor
