# Supplementary material for: Carotenoids and lipid production from Rhodosporidium toruloides cultured in tea waste hydrolysate
Source: Biotechnol Biofuels. 2020 Apr 16;13:74. doi: 10.1186/s13068-020-01712-0 (PMC7161300; doi:10.1186/s13068-020-01712-0)
Supplement: Supplementary file 1 — Additional file 1: Table S1. Sequencing quality evaluation of the cDNA samples of R. toruloides cultured in TWH at different phases. Fig. S1. Examination and verification of the transcriptional levels of the clustered genes with quantitative RT-PCR method. Data were determined through triplicate independent experiments and the error bars represent standard deviation. [file 13068_2020_1712_MOESM1_ESM.doc]

Table S1. Sequencing quality evaluation of the cDNA samples of *R. toruloides* cultured in TWH at different phases.

|  | **Tags sequenced** | **WT**  **(12 h)** | **WT**  **(96 h)** | **RM18**  **(12 h)** | **RM18**  **(96 h)** |
| --- | --- | --- | --- | --- | --- |
| **Number**  **of total tags** | Total tags | 6540521 | 3955217 | 3114845 | **4702778** |
|  | Distinct tags | 340456 | 228917 | 79007 | **28681** |
| **Number**  **of clean tags** | Total tags | 2047211 | 6862034 | 2949103 | **3833790** |
|  | Distinct tags | 22156 | 62034 | 106554 | **66502** |
| **Number of tags matched to gene** | Total tags | 334801 | 703218 | 548049 | **292587** |
|  | Distribution of total tags | 14.05% | 17.73% | 27.23% | **10.48%** |
|  | Distinct tags | 32941 | 38867 | 26793 | **33904** |
|  | Distribution of distinct tags | 22.34% | 46.90% | 38.55% | **34.01%** |
| **Number of**  **tags matched**  **to unique gene** | Total tags | 1646486 | 1726750 | 1503797 | **1159192** |
|  | Distribution of total tags | 50.80% | 43.66% | 48.28% | **24.65%** |
|  | Distinct tags | 18923 | 16539 | 12998 | **10754** |
|  | Distribution of distinct tags | 57.45% | 42.44% | 48.51% | **31.72%** |

**
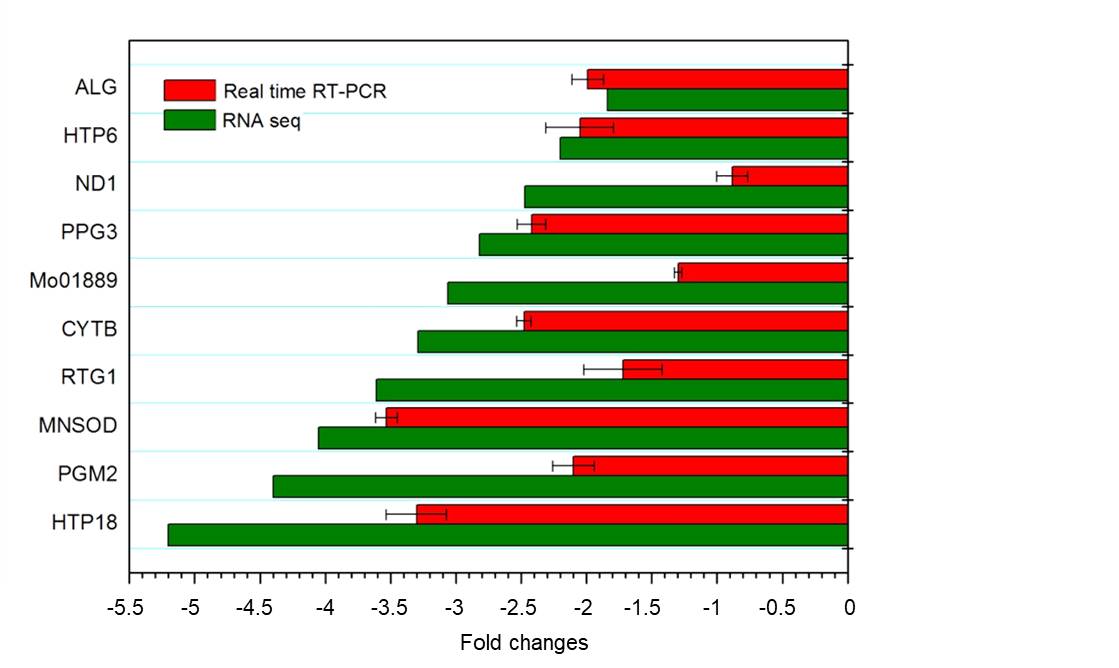

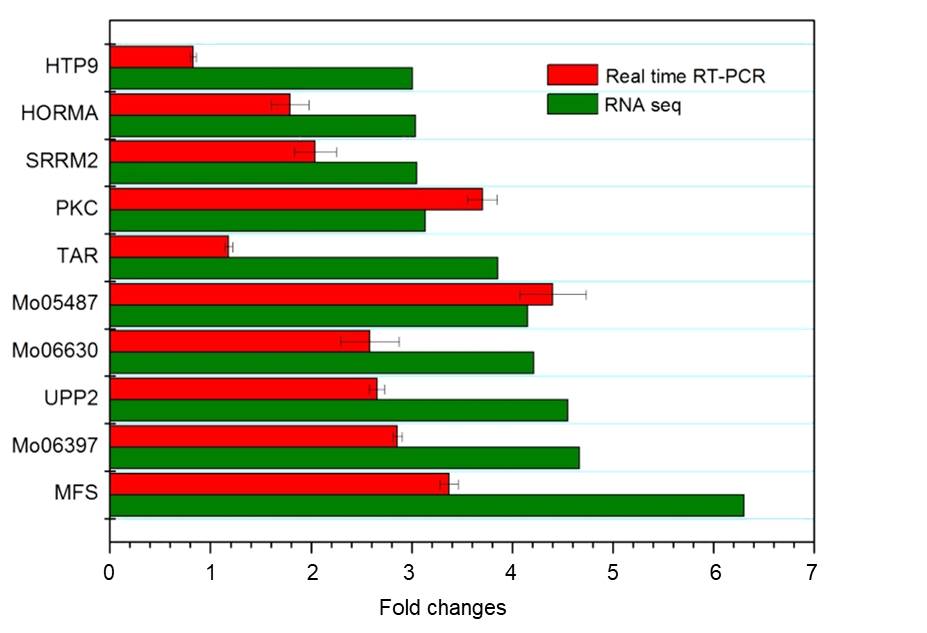
**

Fig. S1 Examination and verification of the transcriptional levels of the clustered genes with quantitative RT-PCR method. Data were determined through triplicate independent experiments and the error bars represent standard deviation.
